# The Lesson Taught by the Epidemic at Plymouth Concerning Typhoid Fever

**Published:** 1885-08-20

**Authors:** M. S. French, E. O. Shakespere

**Affiliations:** Surgeon to the Philadelphia Police Department; Pathologist and Ophthalmic Surgeon of the Philadelphia Hospital; President of the Pathological Society of Philadelphia, etc.


### THE LESSON TAUGHT BY THE EPIDEMIC AT PLYMOUTH CONCERNING TYPHOID FEVER.

By M. S. French, M.D.,
Surgeon to the Philadelphia Police Department,
and E. O. Shakespere, A.M., M.D.,
Pathologist and Ophthalmic Surgeon of the Philadelphia Hospital; President of the Pathological Society of Philadelphia, etc.

That typhoid fever is a specific disease has, in recent years, begun to be generally recognized. Its entire history from inception to termination is that of a disease process separate and distinct from all others. The appreciation of its ætiology is passing through the same phases of doubt and uncertainty which have characterized many other infectious diseases now regarded as specific.

In these days it is almost universally acknowledged that Asiatic cholera, although a disease usually limiting its devastations to localities in which filth and decomposition abound, has its sole cause in one infective agent. But doubt and uncertainty concerning the specific nature of that disease have not been very long removed.

Many respectable authorities still adhere to the opinion that common decomposing or fermenting organic matter is capable of originating typhoid fever *de novo*, yet the weight of authority appears to be drifting toward the belief that this specific disease also is universally caused by one infecting agent, which must enter the human organism before the disease can be produced in man. Most positive facts in support of the latter theory are rapidly accumulating. Reliable observations are numerous, which, in the particular instances related, afford incontestible proof that common fæcal matter had not, in itself, been sufficient to cause the disease. Instances are multiplied where people have for a long time ingested such material with drinking water with impunity—so far as typhoid fever is concerned—until the dejecta from a single patient suffering from this disease have contaminated the water, and caused a sudden outbreak of typhoid fever.

The epidemic of typhoid fever now prevailing at Plymouth, although, perhaps, the most extensive on record (some twelve hundred have been sick and one hundred and thirty have died out of a population of eight thousand), has had its exact minature in other places, but it is not necessary for our present purpose to go into details. It may be well, however, to set forth a summary statement of the circumstances of Plymouth at the time, and before the origin, of the epidemic.

It has been established beyond a reasonable dispute that the hygienic and sanitary surroundings of this town are now, and have been for years, most miserable. Furthermore, it is a fact that water of the Susquehanna river then charged to an unusal degree with the sewage of Wilkesbarre, a city of thirty thousand inhabtants, had been supplied to the people of Plymouth for a week ending fifteen days before the commencement of the epidemic. Yet, notwithstanding these grossly unsanitary conditions, a fair consideration of all the facts bearing upon the cause and origin of this epidemic can, in our opinion, logically lead to no other conclusion than that they alone are not entitled to be regarded as having done more than aided, as secondary factors, some other influence, namely—*the main active cause of the epidemic.*

The scope of this paper will not permit us to dwell upon or enumerate *in extenso* all the facts which support this conclusion. That has already been done in our official report to the Mayor of Philadelphia, and to the relief committee of that city. It is sufficient to mention here the following salient points:

The epidemic is that of genuine typhoid fever, as shown not only by the clinical condition and history of patients examined, but also by a number of autopsies which we were the first to make.

There was no unusual prevalence of typhoid fever in Plymouth, or in the towns around it (including Wilkesbarre), previous to the appearance of the epidemic, and there is not now.

The epidemic began suddenly about the 10th of April of this year and spread with great rapidity, fifty cases occuring daily until five or six hundred of the inhabitants were prostrated.

In the first two or three weeks of the epidemic, those who exclusively used well-water and those who exclusively used river-water escaped the infection. Of children living in houses supplied with well-water, only those who attended the public schools and drank the hydrant-water of the Plymouth Water Company took the disease, while those kept at home went unharmed. It was also noted that those who habitually used beverages other than water were safe from the attack.

On Welsh Hill, a suburb of the town having a population of four or five hundred, mainly supplied by well-water, not one person has been sick of the fever, except a few who frequented the town and drank of the hydrant-water there.

In the village of Broadway, another suburb of Plymouth, containing forty families averaging five members each, there are only two wells, of which the water is considered by the inhabitants to be so unwholesome that it is rarely used for drinking or culinary purposes. The chief water supply there is that of the Susquehanna river, through the pumps and water-mains of the Delaware and Hudson Coal Company, the suction pipe of which extends out upon the bed of the river eighty feet from the shore, and into the main channel at a point *a half mile nearer the sewers of Wilkesbarre* than is the pumping of the water company of Plymouth. In this village not a single case of fever has been found.

In the adjoining village, called Ridge Row, where twenty families live in ten houses, the people are supplied with the same kind of water as are those of Broadway. The one or two cases of fever in this village are with people who had frequented the town of Plymouth and had there drank water from the hydrants. Not a single case exists among those who had exclusively used the Susquehanna or river water.

The facts thus briefly related, in our opinion, conclusively prove that the Susquehanna water, although it was unusually befouled with sewage and other contaminating principles, had nothing whatever to do with the origin and spread of this epidemic, but that, on the contrary, the mountain-water—their usual supply—conveyed to the homes of the people the sole cause of the disease. The circumstances of the case are such that the food supply, including milk, could not possibly have caused this general epidemic.

Now, let us turn to the facts concerning the contamination of that mountain stream. They also have been already related in detail in the report above mentioned. It may be well, however, to state here that it has been shown that the three lower reservoirs on the mountain stream, which nine months of the year supplies Plymouth with water, were on the 20th of March nearly empty; that in a dwelling on the sloping bank of the stream, a little distance above the third reservoir and within seventy feet of the bed of the brook, there was a case of typhoid fever, running its course through January, February, and March; that during most of this period the ground was frozen and covered with snow; that during the illness of this patient the evacuations passed in the night were habitually carried out and thrown upon the snow toward the stream, *no attempt at disinfection having been made*; that about the 25th of March a thaw began, and was followed by slight rains; that on the 26th of March the superientendent of the Plymouth Water Company inspected the reservoirs, and, finding the two upper ones full, that same evening caused the water of the third reservoir to be let down directly to the lowest reservoir; that on the evening of this day pumping from the Susquehanna river ceased, and the town was again entirely supplied from the mountain stream; that thus nearly three months' accumulation of infectious typhoid-fever dejecta was suddenly washed with the melting snow into the brook, and rapidly reached the lower reservoir, and was distributed through the pipes and hydrants of the Plymouth Water Company; that fifteen days after this date the epidemic began; that no other source of unusual pollution of the mountain water was discovered.

In view of all these facts we can arrive at no other conclusion than that the three months' accumulation of infectious dejecta from the one fever patient sick in that mountain-water, was the sole active cause of the epidemic.

The supreme lesson which this unexampled epidemic of typhoid fever should teach to medical men and health officers throughout the world is, in our opinion, threefold:

1. For the production of the specific infectious disease known as typhoid fever, whether individuals or communities are considered, there are required the presence and action of one specific cause, which, elaborated in the intestinal canal of one or more patients suffering with that disease, must be transmitted in an active state to those susceptible. This specific active cause being absent, typhoid fever cannot and does not occur.

2. Epidemics of typhoid fever are a reproach to the communities which they afflict. They are absolutely preventable and controllable, and, from the standpoint of modern experience, neglect to employ proper means to those ends should be regarded as inexcusable.

3. It is the bounden duty of the physician in attendance upon any case of typhoid fever, wherever it may be located, to cause each and every evacuation from the bowels to be *immediately* and *effectually disinfected*, and it is of paramount importance also that the danger of infection of the healthy should be further guarded against by the adoption of efficient means for the destruction of any infectious agent which may exist in the water or in the food. —*N. Y. Med. Journal.*

